# Comparison between adrenal venous sampling and computed tomography in the diagnosis of primary aldosteronism and in the guidance of adrenalectomy

**DOI:** 10.1097/MD.0000000000004986

**Published:** 2016-09-30

**Authors:** Limin Zhu, Ying Zhang, Hua Zhang, Wenlong Zhou, Zhoujun Shen, Fangfang Zheng, Xiaofeng Tang, Bo Tao, Jin Zhang, Xiaohong Lu, Jianzhong Xu, Shaoli Chu, Dingliang Zhu, Pingjin Gao, Ji-Guang Wang

**Affiliations:** a Department of Hypertension; b Department of Radiology; c Department of Urology of Luwan Branch; d Department of Urology; e Shanghai Institute of Hypertension, Shanghai Key Laboratory of Hypertension, Ruijin Hospital, Shanghai Jiaotong University School of Medicine, Shanghai, China.

**Keywords:** adrenal venous sampling, adrenalectomy, age, blood pressure, computed tomography, primary aldosteronism

## Abstract

In our series of patients with primary aldosteronism, we compared diagnostic concordance and clinical outcomes after adrenalectomy between adrenal venous sampling (AVS) and computed tomography (CT) imaging.

Our retrospective analysis included 886 patients with primary aldosteronism diagnosed in our hospital between 2005 and 2014. Of them, 269 patients with CT unilateral adrenal disease were included in the analysis on the diagnostic concordance and 126 patients with follow-up data in the analysis on clinical outcomes after adrenalectomy. Hypertension was considered cured if systolic/diastolic blood pressure (BP) was controlled (<140/90 mm Hg) without medication and improved if BP was controlled with a reduced number of antihypertensive drugs.

In 269 patients with CT unilateral adrenal disease, the overall concordance rate between AVS and CT was 50.5% for lateralization on the same side. The concordance rate decreased with increasing age, with highest rate of 61% in patients aged <30 years (n = 16). In 126 patients with follow-up data after adrenalectomy, the AVS- (n = 96) and CT-guided patients (n = 30) had similar characteristics before adrenalectomy. After andrenalectomy, the AVS-guided patients had a significantly higher serum potassium concentration (4.3 ± 0.3 vs 4.0 ± 0.5 mmol/L, *P* = 0.04) and rate of cured and improved hypertension (98% vs 87%, *P* = 0.03). The AVS-guided patients (n = 50) had slightly higher cured rate than the CT-guided patients (n = 11) in those older than 50 years (26.0% vs 18.2%, *P* = 0.72). The age below which the cured rate in the CT-guided patients was 100% was 30 years.

AVS guidance had moderate concordance with CT and slightly improved clinical outcomes after adrenalectomy. The age below which CT unilateralization achieved 100% cured rate was approximately 30 years.

## 1 Introduction

With the increasing use of aldosterone-to-renin ratio and computed tomography (CT) imaging for screening, the detection of primary aldosteronism has been substantially improved. According to recent reports, the prevalence of primary aldosteronism was as high as 11.2% and 17% to 23% in patients with newly diagnosed^[1]^ or resistant hypertension,^[2–8]^ respectively. In patients with primary aldosteronism, 50% to 70% do not show evidence of a unilateral adenoma or lateralized aldosterone hypersecretion and classified as bilateral adrenal hyperplasia and receive medical treatment. However, 30% to 50% of patients do have unilateral adrenal disease, including aldosterone-producing adenoma and unilateral adrenal hyperplasia and require adrenalectomy.^[9]^ After surgical removal of the gland or the tumor, hypertension might be cured without medication in 29.5%^[10]^ to 31.5%^[11]^ of patients, and blood pressure (BP) control might be improved in an additional 42.3%^[11]^ to 53.0%^[10]^ of patients.

CT imaging is sensitive in the differentiation of adrenal gland lesions, but inaccurate in the determination of functional disorders.^[12,13]^ Current guidance documents therefore recommend adrenal venous sampling (AVS) for the diagnosis of unilateral adrenal disease for surgery except those patients aged less than 40 years, with a typical profile of primary aldosteronism and a clear unilateral adenoma and a normal contralateral adrenal gland on CT imaging.^[14]^ Based on complete concordance (100%) between AVS and adrenal imaging, Lim et al^[15]^ recently proposed that adrenal imaging performed well, and therefore AVS was not needed in patients younger than 35 years. This age-related discrimination on the necessity for AVS was mainly based on the fact that in younger subjects the prevalence of nonfunctional incidentaloma was low^[16]^ and the concordance between CT imaging and AVS was high.^[17]^ There is some but not much evidence from prospective outcome studies on adrenalectomy.^[1,18]^ We therefore investigated our series of patients with primary aldosteronism and compared the diagnostic concordance and clinical outcomes after adrenalectomy between adrenal CT imaging alone and the combination of adrenal CT imaging with AVS.

## 2 Methods

### 2.1 Study designs and population

This retrospective analysis was performed in our series of patients who were diagnosed with primary aldosteronism between May 2005 and September 2014 at the Department of Hypertension, Ruijin Hospital, Shanghai, China. The ethics committee of Ruijin Hospital approved the study protocol. All patients gave written informed consent.

The diagnosis of primary aldosteronism was based on an aldosterone/renin ratio of >240 (ng/dL)/(ng/mL/h) with 2 independent samples and confirmed by either a fludrocortisone test or a saline infusion test. Before and during the work-up, patients were advised to be on a diet of regular salt intake and withdraw mineralocorticoid receptor antagonists for at least 6 weeks; nonpotassium-sparing diuretics for 4 weeks; and β-blockers, angiotensin-converting enzyme inhibitors, and angiotensin II type 1 receptor blockers for 2 weeks. Nondihydropyridine calcium blockers and/or α_1_-blockers were prescribed for BP control, as necessary. Hypokalemia was corrected by oral potassium chloride supplementation.

### 2.2 AVS and CT procedures

All patients first underwent adrenal CT scan (1.25–3.75 mm/slice) for subtyping. The case was considered unilateral if a unilateral radiolucent nodule (<10 Hounsfield units on noncontrast) was detected with a diameter of at least 5 mm and with the remaining ipsilateral and contralateral glands being normal. Patients underwent AVS if they were considered candidates for adrenalectomy according to the clinical manifestations of primary aldosteronism with no contraindication for surgery and were willing to receive the operation. AVS was carried out without cosyntropine stimulation and with subsequent catheterization. A selectivity index (defined as the ratio of adrenal venous plasma cortisol concentration to peripheral plasma cortisol concentration) of ≥3 was considered to indicate correct catheterization. A lateralization index (defined as the ratio of cortisol-corrected aldosterone from dominant side to nondominant side) of ≥2 was considered to indicate lateralization.

### 2.3 Follow-up after adrenalectomy

Adrenalectomy was carried out laparoscopically. Operated patients were followed up either through clinic visits or by telephone interview. The data of the last visit were used for analysis. Hypertension was considered cured if systolic/diastolic BP was <140/90 mm Hg without medication, and improved if BP was <140/90 mm Hg with a reduced number of antihypertensive drugs. Treatment was considered failed if BP was ≥140/90 mm Hg with an increase or no change in the number of preoperative antihypertensive drugs.

### 2.4 Statistical analysis

Statistical analysis was performed using SPSS Statistics 19.0 (Chicago, IL). Differences were considered statistically significant if the *P* value was <0.05. Data were expressed as mean ± standard deviation or median (25th–75th percentile), depending on distribution. Skewed variables were transformed logarithmically or with square transformation. Continuous variables were analyzed using the Student *t* test. Categorical variables were compared using the chi-squared test.

## 3 Results

### 3.1 Flow of patients

**Figure 1:**
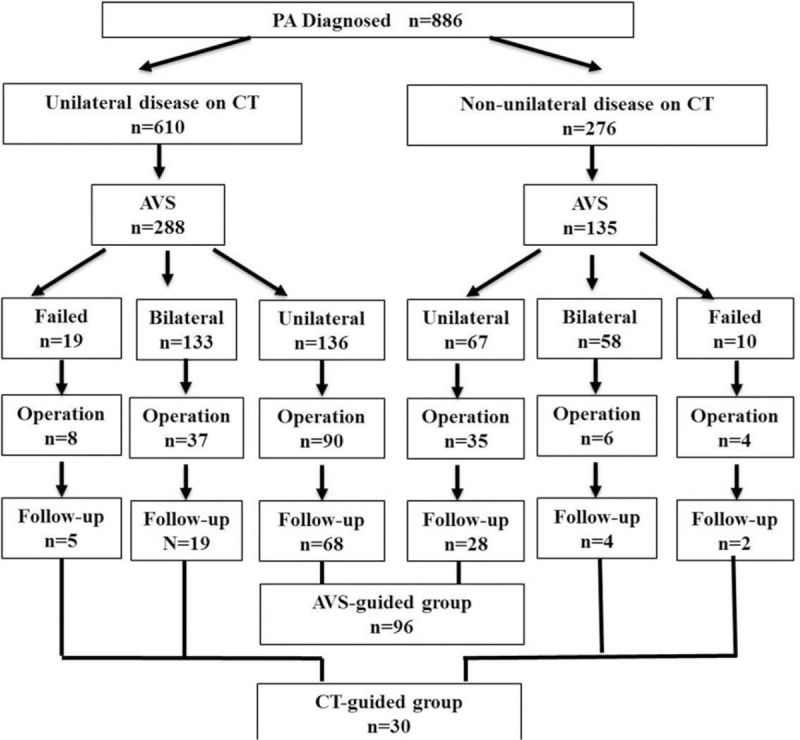
Flow of patients. AVS = adrenal venous sampling, CT = computed tomography.

Our series included 886 patients with primary aldosteronism (Fig. 1). Adrenal CT imaging showed unilateral disease in 610 (68.8%) patients and nonunilateral disease in 276 (31.2%) patients. AVS was performed in 288 (47.2%) and 135 patients (48.9%), respectively, and succeeded in 269 (93.4%) and 125 (92.6%) patients, respectively. Adrenalectomy was performed in 125 and 55 patients as guided by AVS lateralization and CT imaging, respectively. Of these patients, 96 (76.8%) and 30 (54.5%), respectively, were followed up for clinical outcomes.

Of the 55 patients who had adrenalectomy as guided by CT imaging, 45 and 10 patients had unilateral and nonunilateral diseases, respectively (Fig. 1 and Table 1). For the 45 patients with unilateral disease on CT imaging, the reasons for adrenalectomy included persistent hypokalemia after oral supplement of potassium chloride (n = 13), 24-hour urinary aldosterone excretion ≥25 μg (n = 9), adrenal lesion diameter ≥2.0 cm (n = 10) and refractory hypertension (n = 13), and the choice of the adrenal gland to remove was based on the unilateral adenoma presentation. For the 10 patients with nonunilateral disease on CT imaging, the reasons for adrenalectomy included persistant hypokalemia after oral supplement of potassium chloride (n = 3), 24-hour urinary aldosterone excretion ≥25 μg (n = 4), adrenal lesion diameter ≥2.0 cm (n = 1) and refractory hypertension (n = 2), and the choice of the adrenal gland to remove was based on typical adenoma presentation on 1 side (diameter 8–20 mm) and nodular presentation on the opposite side on CT imaging, with a higher adrenal aldosterone/cortisol ratio on the adenoma side.

**Table 1:**
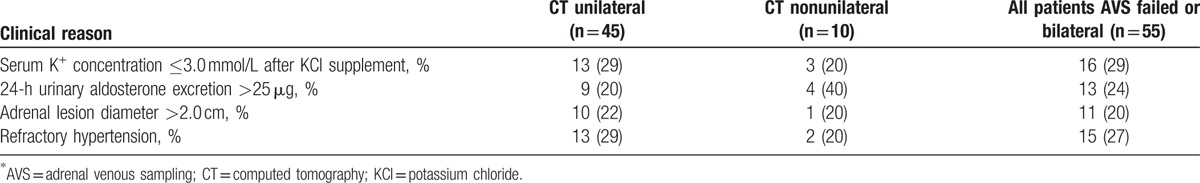
Classification of clinical reasons for adrenalectomy in patients whose adrenal venous sampling failed or showed bilateral disease.

### 3.2 Diagnostic concordance between AVS and adrenal CT imaging

**Figure 2:**
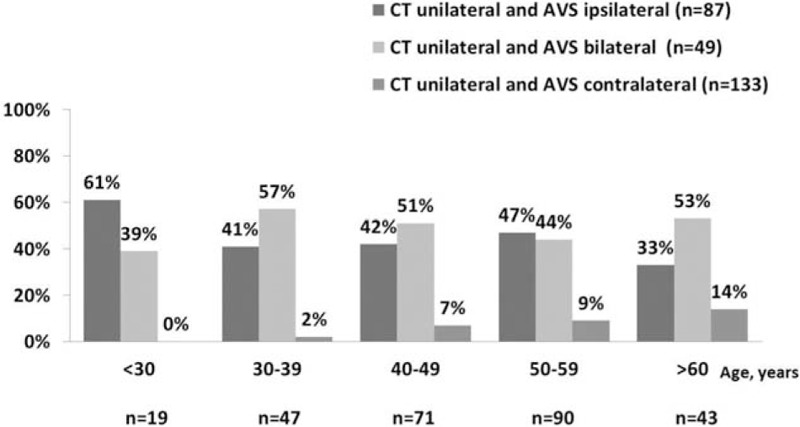
Concordance between adrenal venous sampling (AVS) and computed tomography (CT) according to age in patients with CT unilateral adrenal disease (n = 269). The complete disconcordance (CT unilateral and AVS contralateral) rate was higher with age increasing (*P* = 0.001).

We first studied the diagnostic concordance between adrenal CT imaging and AVS in patients with CT unilateral disease and with successful AVS (n = 269, Fig. 2). Overall, the concordance rate was 50.5%; it decreased with age increasing, with highest rate of 61% in patients younger than 30 years (n = 16). None of the patients younger than 36 years had CT and AVS showing opposite lateralization.

### 3.3 Clinical outcomes after adrenalectomy

**Figure 3:**
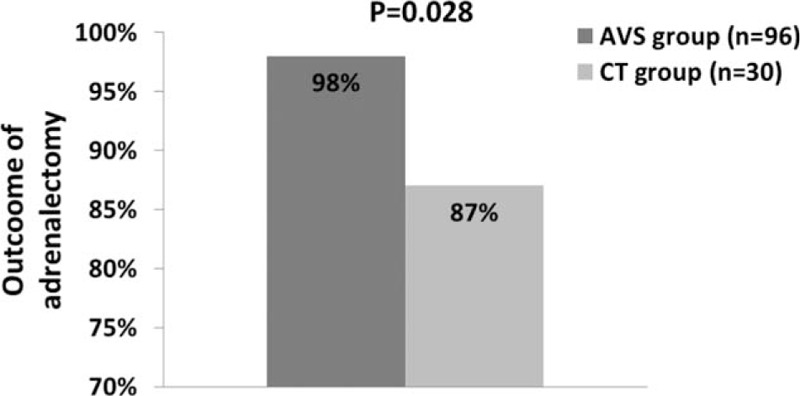
Rate of cured and improved hypertension after adrenalectomy as guided by adrenal venous sampling (AVS) or computed tomography (CT) imaging. The *P* value for the comparison between AVS and CT imaging is given. For the definitions of “cured” and “improved” hypertension, see Section 2.

We then investigated clinical outcomes of adrenalectomy in 96 patients guided by AVS lateralization and 30 patients guided by CT imaging (AVS bilateral n = 23 and AVS failure n = 7, Fig. 1). Table 2 shows the characteristics of patients before adrenalectomy. Before adrenalectomy, patients guided by CT and AVS were similar, except that the AVS-guided patients had lower plasma renin activity (*P* = 0.045) and higher 24-hour urinary aldosterone excretion (*P* = 0.043). After adrenalectomy, the AVS-guided patients had a shorter duration of follow-up than the CT-guided patients (*P* ≤ 0.001). They had similar BP control with a similar number of antihypertensive drugs (Table 3). Fewer patients required the use of spironolactone in the AVS- than CT-guided group (1 vs 3 patients; *P* = 0.04), because of surgical treatment failure. However, the AVS-guided patients had a significantly higher serum potassium concentration (4.3 ± 0.3 vs 4.0 ± 0.5 mmol/L, *P* = 0.04) and lower prevalence of hypokalemia (1.0% vs 10.0%, *P* = 0.04). The total rate of cured and improved hypertension in the AVS group was significantly higher than in the CT group (98% vs 87%; *P* = 0.03, Fig. 3). The AVS-guided patients (n = 50) had slightly higher cured rate than the CT-guided patients (n = 11) in those older than 50 years (26.0% vs 18.2%, *P* = 0.72). Among the CT-guided patients, the 6 patients with nonunilateral disease on CT imaging and with failed (n = 2) or bilateral AVS (n = 4) had similar BP control (3 cured and 3 improved) as the rest of the 24 patients (14 cured, 6 improved, and 4 failed).

**Table 2:**
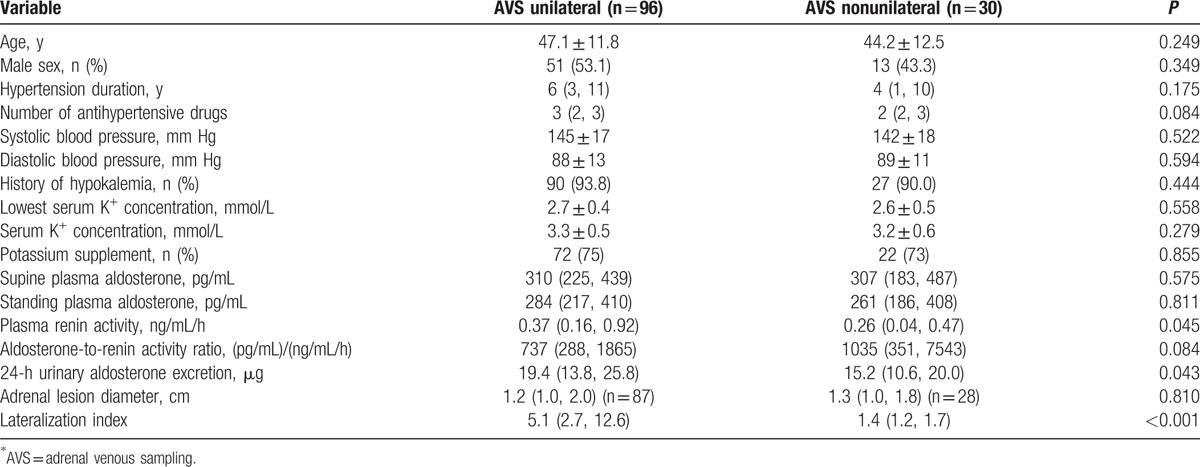
Clinical characteristics before adrenalectomy in patients with follow-up data.

**Table 3:**
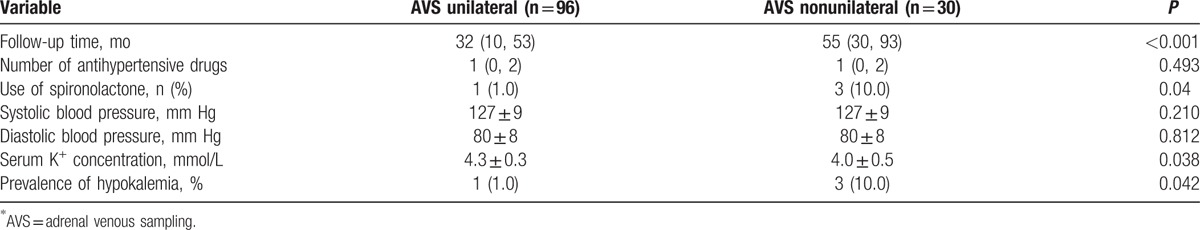
Clinical outcomes after adrenalectomy in patients with follow-up data.

In further analysis in patients younger than 40 years and the AVS-guided patients as comparator, the age below which the cured rate in the CT-guided patients was 100% was 30 years. The corresponding cured rate in the AVS-guided patients was 64%.

## 4 Discussion

Although AVS and adrenal CT imaging had only moderate agreement in patients with CT unilateral adrenal disease, adrenalectomy guided by both techniques improved control of hypertension, with a total cured and improved rate of 87% to 98%. AVS further improved clinical outcomes after adrenalectomy especially in subjects older than 50 years. The age below which the cured rate in the CT-guided patients was 100% was 30 years.

Our finding is in line with the results of a recent study in 263 patients who underwent unilateral adrenalectomy for the treatment of primary aldosteronism at the Mayo Clinic (Rochester, MN).^[15]^ In 143 patients with long-term follow-up data, hypertension was cured in 53 (41.7%) and improved in 59 (46.5%) patients. In 133 patients with long-term follow-up and known cure status of primary aldosteronism, the rate of concordance with the surgically documented side was much higher for AVS (97.1%) than for adrenal imaging with CT or magnetic resonance (58.6%). Nonetheless, adrenal imaging and AVS were 100% concordant in 6 patients younger than 35.1 years. The authors of the study therefore concluded that adrenal imaging performed poor in general but sufficiently well in those younger than 35 years.

The results of several earlier studies also support that AVS may be mandatory for the diagnosis of primary aldosteronism.^[12,19–21]^ In a study of 62 patients with primary aldosteronism, 38 had adrenal CT imaging and successful bilateral AVS. In patients with AVS proven adrenal producing adenoma (n = 15) and bilateral adrenal hyperplasia (n = 21), respectively, only 8 and 4 had concordant findings with CT imaging. The investigators therefore concluded that adrenal CT imaging was not a reliable method, and AVS was essential for the diagnosis of primary aldosteronism.^[19]^ Similar results were observed in a study in 203 patients with primary aldosteronism (age range, 17–80 years; 163 men). AVS was successful in 194 patients (95.6%) and showed unilateral aldosterone hypersecretion in 110 patients (56.7%), including 24 (41.4%) of 58 patients with normal adrenal CT findings, 24 (51.1%) of 47 patients with unilateral micronodule (≤10 mm) apparent on CT (7 had unilateral aldosterone hypersecretion from the contralateral adrenal), 21 (65.6%) of 32 patients with unilateral macronodule (>10 mm) apparent on CT (1 had unilateral aldosterone hypersecretion from the contralateral adrenal), 16 (48.5%) of 33 patients with bilateral micronodules, and 2 (33%) of 6 patients with bilateral macronodules. Overall, if the diagnosis was based on CT findings alone, 42 patients (21.7%) would have missed the opportunity for adrenalectomy and 48 patients (24.7%) might have had unnecessary or inappropriate adrenalectomy.^[12]^ In a retrospective study, CT showed unilateral and bilateral abnormalities, respectively, in 57 and 52 patients with a biochemical diagnosis of primary aldosteronism. Of them, AVS demonstrated concordant results in 46 (80.7%) and 9 patients (17.3%), respectively.^[20]^ Similarly, in a retrospective analysis of 41 patients with primary aldosteronism, unilateral (n = 26) but not nonunilateral lesions on CT (n = 12) had a high positive predictive value of AVS-proven adrenal lesions (85% vs 50%).^[21]^

Our study was not randomized and hence not the best for the comparison of the efficacy between AVS and CT imaging in guiding adrenalectomy. Nevertheless, our study showed that AVS was associated with a slightly but significantly improved clinical outcomes, that is, 99% correction of hypokalemia and 98% improved BP control. However, AVS in general did not increase the cured rate of hypertension in comparison with the CT imaging method. Our finding is in keeping with the results of several previously published studies.^[15,22]^ In the aforementioned study reported by Lim et al,^[15]^ the total rate of cured and improved hypertension was 88.2%. In a study of 13 patients with doubtful lateralization on adrenal imaging, AVS was performed in 8 patients. The AVS-guided adrenalectomy cured the disease biochemically in 100% and cured and improved hypertension in 85% of the patients.^[22]^

Our study provided new evidence on the age limit for the decision of AVS in younger subjects. Our finding suggests that 30 years might be the proper age for not having AVS, as appropriate. Our finding is slightly different from the results of several recent studies^[15,21]^ and the current recommendations.^[14]^ In the aforementioned study from Lim et al,^[15]^ the authors proposed 35 years as the age limit for exemption of AVS according to 100% of concordance rate between AVS and adrenal CT imaging in 6 patients of 35.1 years or younger. In a recent study of 41 patients with primary aldosteronism, 16 were 40 years or younger.^[21]^ Eleven had adrenal CT unilateral lesions. One patient declined surgery. Ten patients had histological confirmation of adenoma. These authors concluded that all patients aged under 40 years with unilateral CT lesions may be directed to surgery without AVS. More and larger sample studies are apparently required to address this important question.

Our study had several limitations. First, although our study included follow-up data after adrenalectomy, it was retrospective. To some extent, because of this retrospective nature, our follow-up was incomplete in terms of the number of patients and the clinical and biochemical data. Second, because of the high exclusion rate, the sample size of our present analysis was relatively small, especially for the age-stratified analysis. Third, in our follow-up study, BP was only measured once in the clinic. Ambulatory or home BP monitoring would be preferred for the evaluation of BP control. Finally and importantly, selection bias was apparent, especially for the CT-guided patients. The CT-guided group included patients whose AVS failed or did not show unilateral aldosterone hypersecretion. Although patients’ wishes played an important part in the decision of surgery, these patients were surgically treated for clinical reasons and after careful lateralization according to the best available imaging and biochemical evidence. The positive outcome results of adrenalectomy suggest that future research should address the issue of unilateral adrenalectomy in patients whose AVS fails (it may fail) and who have bilateral aldosterone hypersecretion, as also indicated by a recent study by Sukor et al^[23]^ on unilateral adrenalectomy in bilateral aldosteronism.

In conclusion, AVS guidance had moderate agreement with CT imaging guidance and was associated with improved clinical outcomes after adrenalectomy. The age below which CT unilateralization achieved 100% of cured rate was 30 years. An implication of our study is that AVS is mandatory in patients with primary aldosteronism for surgery except those very young patients with CT unilateralization.
